# Surgical resection of intracranial cavernous hemangioma located at uncommon location: Clinical presentation and management

**DOI:** 10.3389/fneur.2023.1105421

**Published:** 2023-02-17

**Authors:** Jiuhong Li, Guisheng Zhang, Qiang Ma, Xiang Li, Jiaojiang He

**Affiliations:** ^1^Department of Neurosurgery/Department of Cardiovascular Surgery, West China Hospital of Sichuan University, Chengdu, China; ^2^Department of Neurosurgery, Lhasa People's Hospital, Lhasa, China

**Keywords:** cavernous hemangioma, uncommon location, radiological features, treatment, outcome

## Abstract

**Background:**

Intracranial cavernous hemangiomas (CHs) usually originate from the cerebral and cerebellar hemispheres, while the clinical features and optimum treatment of CHs that originate from atypical locations remain unclear.

**Methods:**

We conducted a retrospective analysis of CHs that originated from the sellar, suprasellar, or parasellar region, the ventricular system, the cerebral falx, or the meninges in patients who underwent surgery from 2009 to 2019 in our department.

**Results:**

In our study, fourteen patients with pathologically confirmed CHs in uncommon locations (UCHs) were enrolled; 5 were located at the sellar or parasellar region, 3 at the suprasellar region, 3 at the ventricular system, 2 at the cerebral falx, and 1 originated from parietal meninges. The most common symptoms were headache and dizziness (10/14); however, none presented with seizures. All UCHs located in the ventricular systems and 2 of the 3 UCHs located in the suprasellar region manifested as hemorrhagic lesions and shared similar radiological features compared with axial CHs; other locations of UCHs did not have a “popcorn” appearance on T2-weighted image. Nine patients achieved GTR, 2 achieved STR, and 3 achieved PR. Four out of five patients who received incomplete resection underwent adjuvant gamma-knife radiosurgery. During the average follow-up of 71.1 ± 43.3 months, no patient died and one patient encountered recurrence and *de novo* formation of midbrain CH. Most patients had an excellent KPS score of 90–100 (9 of 14) or a good KPS score of 80 (1 of 14).

**Conclusion:**

We suggest that surgery is the optimum therapeutic method for UCHs located at the ventricular system, dura mater, and cerebral falx. Stereotactic radiosurgery plays an important role in the treatment of UCHs located at the sellar or parasellar region and remnant UCHs. Favorable outcomes and lesion control could be achieved by surgery.

## Introduction

Intracranial cavernous hemangiomas (CHs), also called cavernous malformations, have an estimated incidence of 0.4–0.8% of the population (1). They typically locate in the cerebral hemispheres, cerebellar hemispheres, and brainstem, while CHs in uncommon locations (uncommon cavernous hemangioma, UCH), which locate at the sellar or parasellar region, the suprasellar region, the ventricular system, and originate from meninges, are rare (2, 3). UCHs manifest differently in many aspects, such as clinical presentation, radiological features, and radiation sensitivity, compared with their parenchymal counterparts (4–6). Correct preoperative diagnosis of UCHs is challenging because of fewer common locations from which CHs seldom arise as well as the atypical radiological features.

Due to their rarity, most previous studies of UCHs have been case reports or small case series (7–13). Therefore, neither clinical features nor the optimum treatment of UCHs have been fully discussed.

Therefore, in the present study, we retrospectively analyzed the clinical features, radiological findings, management, and outcomes of 14 patients with histologically confirmed UCHs in our hospital. To the best of our knowledge, this is the first study reporting large case series of all intracranial UCHs.

## Materials and methods

We conducted a retrospective analysis on surgically treated UCHs in our institution. The inclusion and exclusion criteria were as follows:

### Inclusion criteria

(1) Patients underwent surgery or biopsy between December 2009 and December 2019 in our department;

(2) All lesions had to be pathologically confirmed cavernous hemangiomas;

(3) The lesions were intracranial masses;

(4) Lesions originating from the sellar, suprasellar, or parasellar region, the ventricular system, the cerebral falx, or the meninges were included.

### Exclusion criteria

(1) Cavernous hemangiomas located at and originating from the cerebral hemispheres, the basal ganglia region, the brainstem, or the cerebellum were excluded;

(2) Cavernous hemangiomas located at the orbital apex were excluded;

(3) Cavernous hemangiomas originating from the scalp or the skull were excluded;

(4) Follow-up times <6 months were also excluded.

The clinical and radiological findings, optimum treatment, and prognosis were discussed. Data, including patient age, sex, symptoms, and durations of symptoms before diagnosis, were obtained. All the patients were evaluated with Gd-enhanced cranial magnetic resonance imaging (MRI) scan preoperatively. Location, size (the maximum diameter of the lesion), and enhancement pattern were noted. The intraoperative bleeding volume was recorded. The treatment-related factors including the extent of surgical resection (gross total resection [GTR] [>90% resection], subtotal resection [STR] [<90% resection], partial resection [PR] [<50% resection], or biopsy only), and radiation treatment were recorded from medical records. Follow-up was performed at 3, 6 months, and then once a year after surgery. During the follow-up period, the progression of residual lesions, lesion recurrence, and metastasis were monitored using postoperative Gd-enhanced MRI. The neurological functional outcomes were assessed using the Karnofsky performance scale (KPS).

Student's *t*-test was used to compare continuous variables. A *P*-value of < 0.05 was considered significant. The analyses were performed using SPSS, version 23.0 (IBM Corp., Armonk, New York, USA).

## Results

### Patient sample and clinical features

From December 2009 to December 2019, 241 patients with pathologically confirmed intracranial CHs and 14 (5.8%) patients with UCHs underwent surgery at our department. Patient characteristics and clinical features are summarized in Supplementary Table 1. Of the 14 patients, 8 were women and 6 were men with a mean age of 39.8 ± 18.0 years (range, 6–68 years), which was slightly higher compared to total CHs (age, 35.5 ± 14.7 years; *p* = 0.389). The most common symptoms were headache and dizziness (*n* = 10). Other symptoms and signs included impaired vision (*n* = 4), fever (*n* = 2), vomiting (*n* = 1), weight gain (*n* = 1), and sensory disturbance (*n* = 1). The mean symptom duration was 3.3 months (range, 1 day to 24 months).

### Radiological features

**Figure 1:**
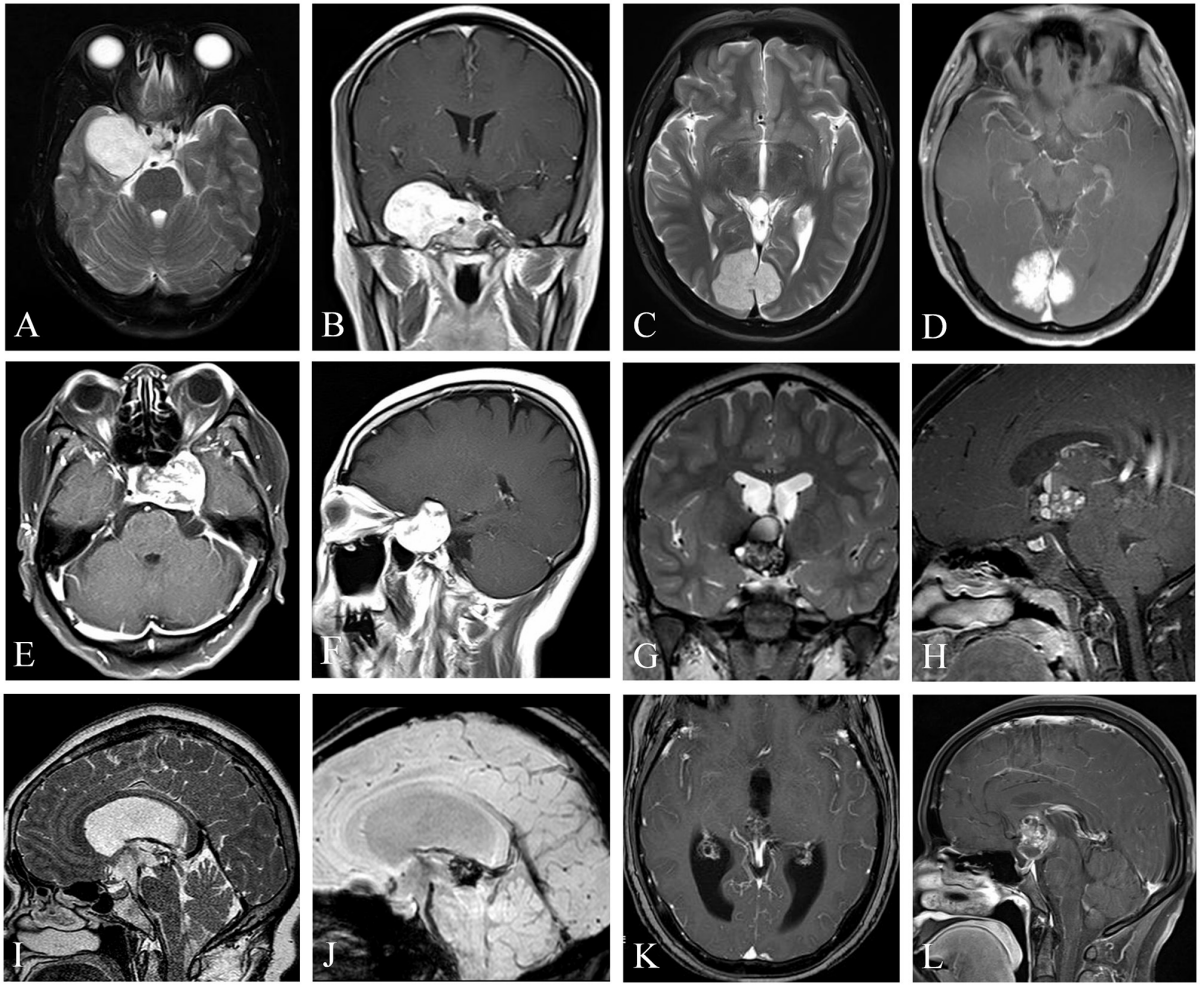
Uncommon cavernous hemangiomas (UCHs) with different locations and radiological features. Case 3, a right enhanced parasellar region CH with pituitary fossa involvement and dural tail sign **(A, B)**, mimicking a sphenoid ridge meningioma. Case 12, a strongly enhanced CH originating from occipital falx was depicted with a dural tail sign **(C, D)**, masquerading as a meningioma. Case 4, a CH located at the sellar or parasellar region, mimicking a meningioma **(E, F)**. Case 10, a CH located in the third ventricle, with classic popcorn-like radiological features, with a hypointense rim on T2-weighted image **(G)** and heterogenous enhancement **(H)**. Case 11, a CH located at the inlet of midbrain aqueduct **(I–K)**, with susceptibility-weighted imaging **(J)** showing hemosiderosis deposition of the lesion, indicating a diagnosis of a CH. Case 6, a CH located at the suprasellar region mimicking a craniopharyngioma **(L)**. **(I–K)** Demonstrated in Neurosurgical Focus Video—*Anterior endoscopic transcortical approach to a pineal region cavernous hemangioma*.

The radiological features are summarized in Supplementary Table 2. Of the 14 patients, 5 UCHs were located at the sellar or parasellar region, 3 at the suprasellar region, 3 at the ventricular system, 2 at the cerebral falx, and 1 originated from parietal meninges. On the T1-weighted image, eight cases showed hypointensity, 4 cases had hypo-hyper intensity, and 2 cases had hypo-iso intensity. On the T2-weighted image, 8 cases revealed hyperintensity, 3 cases had hypo-hyper intensity, 1 case had iso-hyper intensity, 1 case had hypo-iso intensity, and 1 case had hyperintensity. Most (*n* = 11, 78.6%) cases demonstrated heterogeneous enhancement after the administration of gadolinium, while only 3 cases demonstrated homogenous enhancement (Figure 1). Hemorrhage history or radiological diagnosis of hemorrhagic lesion was detected in 7 patients, including 3 in the suprasellar region (Cases 6–8), 1 in the third ventricle (Case 10), 1 in the septum pellucidum (Case 9), 1 in the midbrain aqueduct (Case 11), and 1 in the cerebral falx (Case 13). Preoperative diagnosis was cavernous hemangioma in only 4 cases, meningioma in 6 cases, craniopharyngioma in 2 cases, pituitary adenoma in 1 case, and angioma in 1 case (Supplementary Table 2).

### Treatment

Of the 14 patients, 9 underwent microsurgical resection and 5 underwent endoscopic surgical resection. Nine patients achieved GTR, 2 achieved STR, and 3 achieved PR. Of the five patients, four underwent adjuvant gamma-knife radiosurgery and received incomplete resection. The median intraoperative bleeding volume was 100 ml (range, 20–3,000 ml).

### Outcomes and prognosis

**Figure 2:**
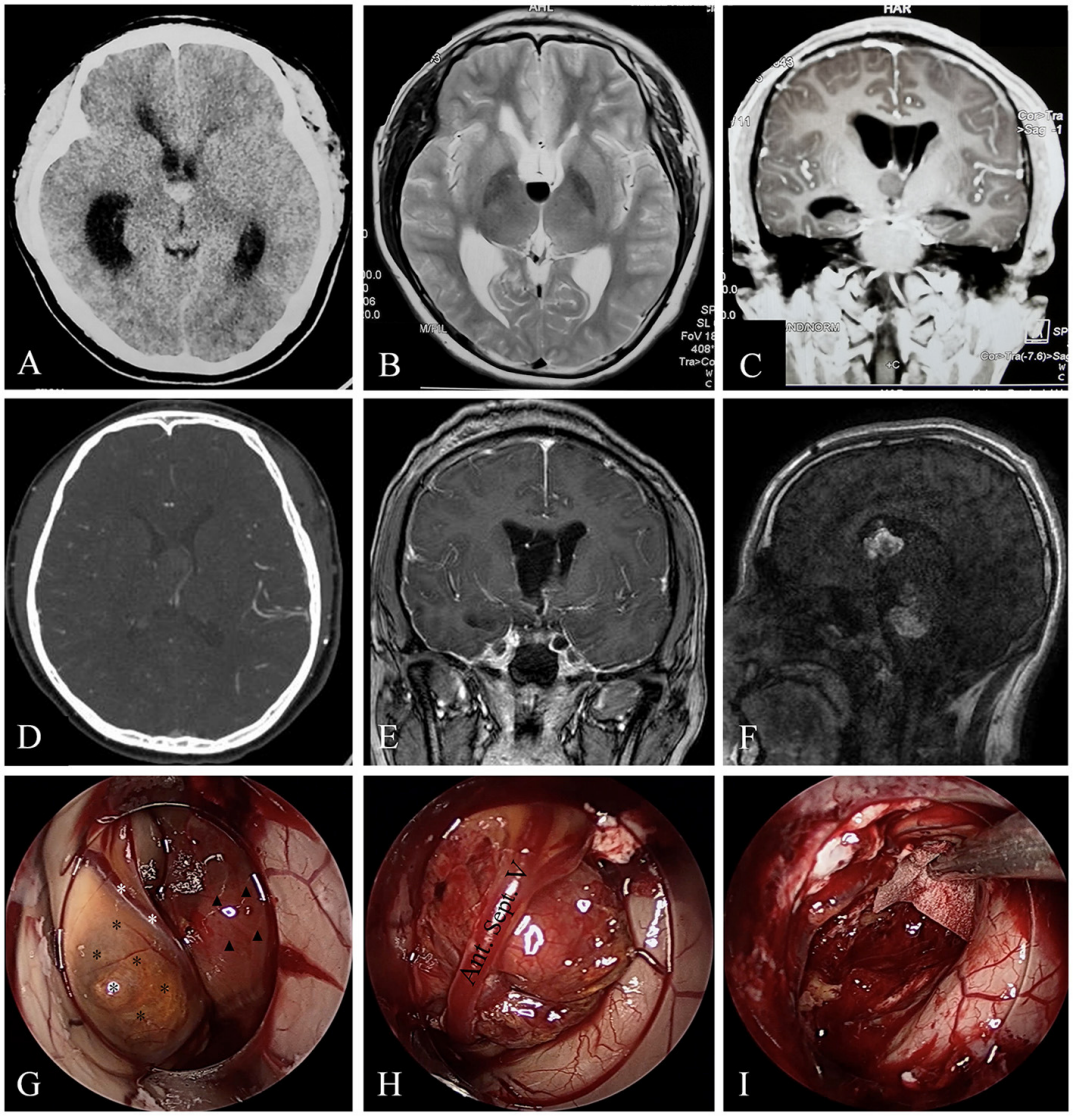
A rare case of CH originating from septum pellucidum with multiple recurrences. Case 9, the brain CT **(A)** of a 16-year-old boy revealed a hemorrhagic lesion of the third ventricle. T2-weighted image **(B)** showed fluid level in the lesion, suggesting hemorrhage. Coronal enhanced MRI **(C)** depicted a non-enhanced lesions causing obstructive hydrocephalus. Brain CTA **(D)** in our hospital revealed the lesion was without apparent blood supply. Endoscopic surgical resection was achieved in our hospital, and postoperative enhanced MRI showed gross total resection of the lesion **(E)**. Gd-enhanced MRI **(F)** showed the two lesions were heterogeneously enhanced. Intraoperative images **(G–I)** revealed the lesion (black asterisks) originated from the septum pellucidum, compressing the anterior septum vein (white asterisks). A capsuled hematoma (triangles) could also be observed in the third ventricle **(G)**. We carefully separated and protected the anterior septum vein **(H)** during the operation. At last, the lesion and hematoma were removed **(I)**.

One patient suffered from intracranial infection postoperatively (Case 5), and 1 patient endured hypopituitarism and recovered 3 months after surgery (Case 4). Most patients experienced improvements in their symptoms, and 3 patients had stable vision impairment (Cases 1, 6, and 7). Apart from Case 4, no new neurological disorder or deficit was detected postoperatively. Most patients had an excellent KPS score of 90–100 (9 of 14) or a good KPS score of 80 (1 of 14). However, 1 patient (Case 9) experienced slurred speech and left lower limb weakness (KPS score, 60) and 3 patients (Cases 1, 6, and 7) suffered from impaired or blurred vision (KPS score 60–70). During an average follow-up of 71.1 ± 43.3 months, no patient died and one patient (Case 9) encountered recurrence and *de novo* formation of CH (Figure 2); however, this patient refused further treatment. For those who received GKRS after incomplete resection, no recurrence or *de novo* formation was detected.

## Case illustration

### Case 9

A 16-year-old adolescent boy presented with headache, fever, and vomiting in another hospital for 20 days. Brain computed tomography (CT) and magnetic resonance imaging (MRI) revealed a hemorrhagic lesion of the third ventricle, causing obstructive hydrocephalus (Figures 2A–C). Brain computed tomography angiography (CTA) revealed that the lesion was without apparent blood supply (Figure 2D). Lumbar puncture testing showed an increased cerebral spinal fluid (CSF) pressure (18 cm of water column), and CSF analysis revealed elevated nucleated cells of 140 ^*^ 10^6^/L and a trace protein of 1.39 g/L, and no tumor cells were detected. Serum alpha-fetoprotein (AFP) and β-HCG results were normal. A transfrontal endoscopic surgery was adopted to remove the mass. The mass was gray and yellowish, with remote hemorrhage originating from the septum pellucidum (Figures 2G–H) and blocking the interventricular foramen, and it was removed completely (Figure 2I). The intraoperative bleeding volume was approximately 100 ml. A postoperatively enhanced MRI (Figure 2E) confirmed the GTR of the lesion. The pathological diagnosis was cavernous hemangioma. The symptoms were completely alleviated. After 10 months, however, he presented with a sudden headache, slurred speech, and left limb hemiparesis for 5 days. Enhanced MRI depicted a recurrence of the hemorrhagic lesion of the third ventricle as well as a *de novo* midbrain hemorrhagic lesion (Figure 2F). We recommended surgical resection of the multiple CHs and performing a genetic analysis, but the patient and his guardians refused and got discharged. The patient was still alive 6 months later, and symptoms alleviated compared with the last discharge.

## Discussion

**Figure 3:**
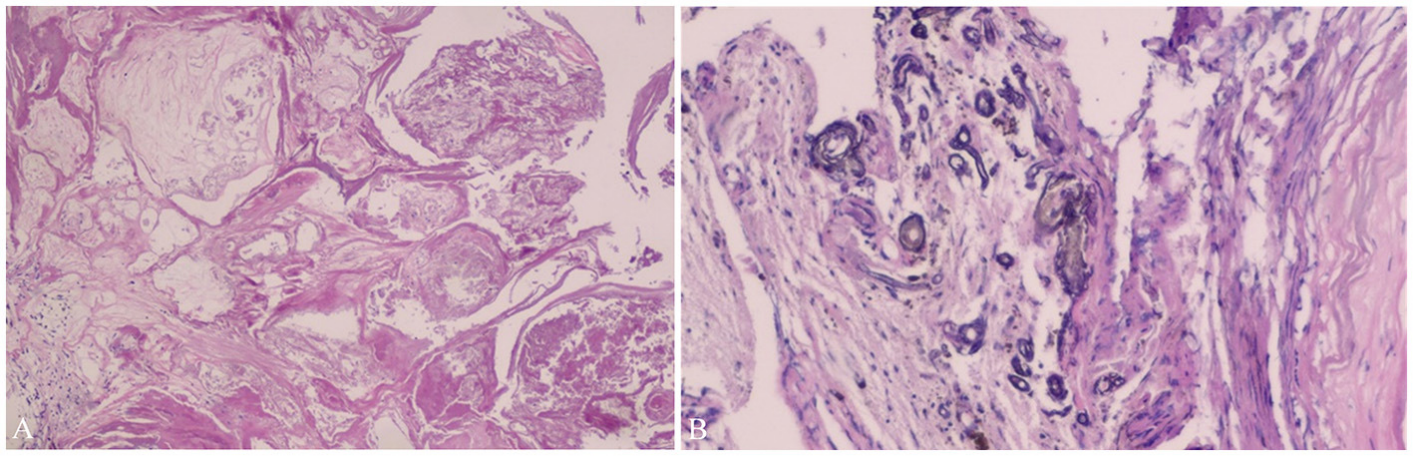
Pathological findings of Case 11. Pathological findings (Case 11) show vascular tissues of different sizes **(A)** and calcification **(B)**, which confirm the diagnosis of cavernous hemangioma (hematoxylin–eosin staining × 20). It has been demonstrated in Neurosurgical Focus Video—*Anterior endoscopic transcortical approach to a pineal region cavernous hemangioma*.

Cavernous hemangiomas, also called cavernous malformations, cavernous angiomas, or cavernomas, are congenital vascular malformations that are the second most common vascular lesions, accounting for 5–15% of all vascular malformations (14). The majority of CHs locate at and originate from the parenchymal tissue, and UCHs are rare, accounting for <10% of all intracranial CHs (12, 13). Radiologically, parenchymal CHs (PCHs) frequently manifest as significant T2 hypointensity, producing a black “halo” around the lesion or “popcorn” appearance on T2-weighted imaging because of peripheral hemosiderin (15, 16) and heterogeneously enhancement on Gd-enhanced MRI. Pathologically, UCHs located at the ventricular system and the suprasellar region tend to share similar histopathological findings in hematoxylin–eosin staining (Figure 3). However, for UCHs located at the sellar or parasellar region and meninges, rich fibrous tissue may be found in hematoxylin–eosin staining (6, 17). Nevertheless, UCHs tend to behave differently compared with PCHs, which causes a diagnostic dilemma. Because of their rarity, the clinical features, treatment strategies, and outcomes of UCHs remain unclear. In our case series, three cases were previously reported as a single case report (6, 17) or video article (18). However, we believe that it would be significant to conduct an updated study focusing on UCHs and report our additional 11 cases of UCHs, which is of potential interest to the neurological community.

### Clinical features

According to our institutional data, UCHs have a slight female predominance (1.33:1) compared with their parenchymal counterparts (1.15:1). No specific symptoms were associated with UCHs, and the symptoms varied by lesion location. The most common presenting symptoms were headache and dizziness (71.4%), while the most common symptom in parenchymal CHs was a seizure (23–79%) (14). Notably, none of our patients presented with seizures. According to the literature, a seizure is a common symptom for dural-based CHs as 6 out of 24 (25%) patients present with epilepsy (10). For CHs located in the ventricular system, the incidence of seizure presentation ranged from 2.9 to 14% (7, 12, 19, 20). None of the patients with CHs in the sellar or parasellar region presented with epilepsy. This discrepancy is not difficult to explain because the seizure was considered to be most likely caused by gliosis and inflammation of parenchyma induced by recurrent microhemorrhage irritation of cerebral CHs (21), which is less likely to be caused by UCHs. According to our institutional data, the mean age of the patients with UCHs was higher than PCHs but did not reach a significant difference.

### Radiological features

The correct preoperative diagnosis rate of CHs in UCHs is low. The most common misleading preoperative diagnosis is meningioma (6 cases). For UCHs originating from the cerebral falx, the dura mater, and the sellar or parasellar region, they could masquerade as meningiomas. No hypointense “halo” ring or “popcorn” appearance was detected in these patients on the T2-weighted image. Furthermore, the dural tail sign was detected on Gd-enhanced MRI in 4 out of 6 of these patients (Figures 1B, D, E) (6, 17), which caused a diagnostic dilemma. Thus, when dealing with dural lesions with the dural tail sign, a possible diagnosis of CHs should at least be reminded for neurosurgeons. UCHs located at the sellar region could masquerade as pituitary adenomas, while UCHs located at the third ventricle or suprasellar region may mimic craniopharyngiomas. Thus, we conclude that, although rare, CHs should be considered as a differential diagnosis for lesions located in the sellar or suprasellar region or third ventricle. Most (71.4%) hemorrhagic UCH lesions are located in the ventricular system (3/7) and in the suprasellar region (2/7); other locations include the sellar region (1/7) and the cerebral falx (1/7). UCHs located at the ventricular system and suprasellar region tend to have a high rate (100% and 67%) of “halo” ring or “popcorn” appearance on T2-weighted image (Figures 1G–K), and hemosiderosis deposition could be detected on susceptibility-weighted imaging (SWI) (Figure 1J), which demonstrates the presence of hemorrhage within the lesion. This finding is similar to the literature (19). We conclude that UCHs located in the ventricular system share similar radiological features with PCHs, and further examinations such as SWI may be considered to facilitate differential diagnosis.

### Treatment strategy

Surgery remains an optimal treatment for UCHs located in the ventricular system or originating from the dura mater and an important therapeutic method for UCHs located in the sellar, suprasellar, or parasellar region. Microscopic or endoscopic alternatives and operative approaches must be tailored to the individual lesion characteristics (12, 22). Microscopic approaches are suitable in most UCHs, while neuroendoscopy could be considered as an alternative for surgical resection of UCHs located at the lateral ventricles, the third ventricles, or the sellar or parasellar region (12, 17, 22). UCHs originating from the septum pellucidum are very rare, and ventricular vein protection during operation is crucial (Figures 2G, H). For UCHs located at the back side of the third ventricle and accompanied by hydrocephalus, we recommend an anterior endoscopic transcortical approach, which could achieve endoscopic third ventriculostomy, alleviating and preventing hydrocephalus due to postoperative adhesion and resection of the lesion at the same time (18). For UCHs, total resection should be considered. In our case series, GTR was achieved in most of the cases (*n* = 9, 64.3%). However, total resection could not be achieved in some cases for certain reasons, especially when severe intraoperative bleeding was encountered. In our case series, 4 of 5 UCHs located at or originating from the sellar or parasellar region had severe intraoperative bleeding, with an intraoperative volume ranging from 800 to 3,000 ml, hindering us from GTR. We conclude that, if a sellar or parasellar region hemangioma is confirmed during surgery and intraoperative bleeding is severe at the same time, an incomplete resection combined with adjuvant gamma-knife radiosurgery is acceptable. Beitch et al. (10) reviewed 23 cases of dural-based CHs, and none of these patients received radiation therapy. They suggest that surgical resection is the gold treatment and that no adjuvant radiosurgery therapy is necessary. We considered that it is liable to achieve total resection in dural-based CHs because they are frequently well circumscribed and without a rich blood supply.

Some researchers suggest that, for CHs located at the sellar or parasellar region, stereotactic radiosurgery (SRS) should be recommended as the optimal treatment (23). SRS is considered effective in lesion control for CHs located in these locations as they are radiation sensitive (5, 23). In contrast, SRS could avoid the risk of pituitary gland impairment, severe cavernous sinus bleeding, and injury to the optic nerve, the oculomotor nerve, the trochlear nerve, and the abducent nerve, among others, that surgery could bring about. Furthermore, for those UCHs with incomplete resection, we recommend gamma-knife radiosurgery (GKRS). In our case series, adjuvant GKRS was adopted in 4 out of 5 UCHs that had an incomplete resection. After a follow-up time of 32–127 months, no recurrence or progression occurred, which is similar to the literature (17, 23–25). Thus, GKRS is a safe and effective therapeutic strategy for lesion control in CHs located at the sellar or parasellar region. In summary, our case series indicated that UCHs are most likely sensitive to radiation, which is different from PCHs, and SRS plays an important role for UCHs located at the sellar or parasellar region and remnant UCHs.

### Prognosis

Our case series revealed that most patients with UCHs who received surgery had a favorable KPS score (≥80) during follow-up, which is similar to the literature (19, 26). The symptoms and signs of most patients with UCHs had improved or became stable after surgery; however, deterioration of symptoms could occur (7, 10, 12, 13, 19, 20, 23, 26, 27). For UCHs located in the fourth ventricle with partial resection, they were at a higher risk of having a poor outcome (7). Postoperative complications included cranial nerve (CN) III, CN VI palsy (26, 28), CN VII paresis (20, 28), visual disorder (29, 30), hydrocephalus (19, 31), cerebellar dysfunction (29), mild hemiparesis (19, 20, 22, 29), hypopituitarism (32), and coma (33). The recurrence rate is relatively low in extra-axial CHs. Li et al. (26) reported a 2.1% recurrence/rebleeding rate for extra-axial CHs located at the parasellar region. However, CHs located in the ventricular system had a higher rate of recurrence/rebleeding. In our case series, one case (Case 9) suffered from recurrence and *de novo* brainstem CHs 10 months after the first operation (Figure 2F). The young age and *de novo* brainstem CH formation may suggest a genetic disorder in the patient (34); however, genetic analysis was refused. Kivelev et al. (20) reported a high recurrence or rebleeding rate (5/12) for intraventricular UCHs. The death rate was 3.8–11.1% in UCHs located at the ventricular system (7, 12, 19, 20) and 2.1% in the parasellar region (26). No death was reported in the sellar region and was dural-based in patients with UCHs.

## Limitations

There are several limitations to our study. First, our study was retrospective research with limitations inherent to the study design. Second, due to the rarity of UCHs, it was difficult to get a meaningful assessment based on the institutional series. Finally, genetic analysis and molecular testing could not be achieved in our case series because of the limitations of the financial situation of patients and medical insurance policy. Therefore, further investigations, in particular large sample sizes and genetic analysis of multicenter prospective studies of this rare lesion subset, are required.

## Conclusion

Cavernous hemangiomas could be located in the entire central nervous system, including extra-axial places. With headache and dizziness being the most common symptoms, UCHs have a low rate of seizure and hemorrhage. UCHs located at the ventricular system and the suprasellar region share similar radiological features with parenchymal CHs, while dural-based UCHs and UCHs originating from the sellar or parasellar region behave differently compared with parenchymal counterparts. Surgery is the optimum therapeutic method for UCHs located at the ventricular system, the dura mater, and the cerebral falx. Stereotactic radiosurgery plays an important role in the treatment of UCHs located at the sellar or parasellar region and remnant UCHs. Favorable outcomes and lesion control could be achieved by surgery.

## Data availability statement

The raw data supporting the conclusions of this article will be made available by the authors, without undue reservation.

## Ethics statement

The studies involving human participants were reviewed and approved by Ethics Committee of West China Hospital of Sichuan University. Written informed consent to participate in this study was provided by the participants' legal guardian/next of kin. Written informed consent was obtained from the individual(s), and minor(s)' legal guardian/next of kin, for the publication of any potentially identifiable images or data included in this article.

## Author contributions

JL and JH: conception and design. JL, GZ, QM, and XL: development of methodology, manuscript writing, review, and revision. JL, GZ, QM, and JH: acquisition and analysis of data. GZ and JH: technical and material support. XL and JH: study supervision. All authors have read and approved the manuscript. All authors contributed to the article and approved the submitted version.

## References

[1] Tian KB Zheng JJ Ma JP Hao SY Wang L Zhang LW. Clinical course of untreated thalamic cavernous malformations: hemorrhage risk and neurological outcomes. J Neurosurg. (2017) 127:480–91. 10.3171/2016.8.JNS16934 27834594

[2] Xia C Zhang R Mao Y Zhou L. Pediatric cavernous malformation in the central nervous system: report of 66 cases. Pediat Neurosurg. (2009) 45:105–13. 10.1159/000209284 19307744

[3] Moran NF Fish DR Kitchen N Shorvon S Kendall BE Stevens JM. Supratentorial cavernous haemangiomas and epilepsy: a review of the literature and case series. J Neurol Neurosurg Psychiatry. (1999) 66:561–8. 10.1136/jnnp.66.5.561 10209164 PMC1736368

[4] Shibata S Mori K. Effect of radiation therapy on extracerebral cavernous hemangioma in the middle fossa. Report of three cases. J Neurosurg. (1987) 67:919–22. 10.3171/jns.1987.67.6.0919 3681431

[5] Gonzalez LF Lekovic GP Eschbacher J Coons S Porter RW Spetzler RF. Are cavernous sinus hemangiomas and cavernous malformations different entities? Neurosurg Focus. (2006) 21:e6. 10.3171/foc.2006.21.1.7 16859259

[6] Wang X Liu JP You C Mao Q. Convexity dural cavernous haemangioma mimicking meningioma: a case report. Br J Neurosurg. (2016) 30:345–7. 10.3109/02688697.2015.1096904 26449587

[7] Kaye J Zeller S Patel NV Herschman Y Jumah F Nanda A. Presentation, surgical management, and postoperative outcome of a fourth ventricular cavernous malformation: case report and review of literature. World Neurosurg. (2020) 137:78–83. 10.1016/j.wneu.2020.01.185 32028002

[8] Simonin A Passaplan C Sancho S Rusconi A Otten P. Giant extra-axial cavernous angioma of the falx: case report. Neurosurgery. (2019) 84:E211–E4. 10.1093/neuros/nyy080 30203083

[9] Entezami P Adamo MA. Rare pediatric presentation of a cavernous angioma of the septum pellucidum. Pediat Neurosurg. (2019) 54:147–9. 10.1159/000496394 30731472

[10] Bteich F Kassab C El Hage G Moussa R Abadjian GA Bou-Nassif R. Atypical presentation of parietal convexity dural-based cavernous hemangioma: a case report and review of literature. World Neurosurg. (2019) 128:403–7. 10.1016/j.wneu.2019.04.119 31009776

[11] Das S Ang LC Ramsay D. Intrasellar cavernous hemangioma presenting as pituitary adenoma: a report of two cases and review of the literature. Clin Neuropathol. (2018) 37:64–7. 10.5414/NP301012 29189199

[12] Shirvani M Hajimirzabeigi A. Intraventricular cavernous malformation: review of the literature and report of three cases with neuroendoscopic resection. J Neurol Surg A Cent Eur Neurosurg. (2017) 78:269–80. 10.1055/s-0036-1594235 28068754

[13] Dorner L Buhl R Hugo HH Jansen O Barth H Mehdorn HM. Unusual locations for cavernous hemangiomas: report of two cases and review of the literature. Acta Neurochirurgica. (2005) 147:1091–6; discussion 6. 10.1007/s00701-005-0567-6 16052290

[14] Washington CW McCoy KE Zipfel GJ. Update on the natural history of cavernous malformations and factors predicting aggressive clinical presentation. Neurosurg Focus. (2010) 29:E7. 10.3171/2010.5.FOCUS10149 20809765

[15] Mokin M Agazzi S Dawson L Primiani CT. Neuroimaging of cavernous malformations. Curr Pain Headache Rep. (2017) 21:47. 10.1007/s11916-017-0649-1 29030748

[16] Vilanova JC Barceló J Smirniotopoulos JG Pérez-Andrés R Villalón M Miró J. Hemangioma from head to toe: MR imaging with pathologic correlation. Radiogr Rev Publ Radiol Soc N Am Inc. (2004) 24:367–85. 10.1148/rg.242035079 15026587

[17] Lan Z Richard SA Li J Xu J You C A. giant solid cavernous hemangioma mimicking sphenoid wing meningioma in an adolescent: a case report. Medicine. (2018) 97:e13098. 10.1097/MD.0000000000013098 30383694 PMC6221700

[18] Li J He J Liu L Zhou L. Anterior endoscopic transcortical approach to a pineal region cavernous hemangioma. Neurosurg Focus Video. (2021) 5:V15. 10.3171/2021.4.FOCVID215 36284913 PMC9549988

[19] Wang C Zhao M Deng X Wang J Shu Q Jiang Z. Neurosurgical management of cavernous malformations located at the foramen of Monro. Neurosurg Rev. (2018) 41:799–811. 10.1007/s10143-017-0930-0 29199381

[20] Kivelev J Niemela M Kivisaari R Hernesniemi J. Intraventricular cerebral cavernomas: a series of 12 patients and review of the literature. J Neurosurg. (2010) 112:140–9. 10.3171/2009.3.JNS081693 19408982

[21] Awad I Jabbour P. Cerebral cavernous malformations and epilepsy. Neurosurg Focus. (2006) 21:e7. 10.3171/foc.2006.21.1.8 16859260

[22] Woodall MN Catapano JS Lawton MT Spetzler RF. Cavernous malformations in and around the third ventricle: indications, approaches, and outcomes. Oper Neurosurg. (2020) 18:736–46. 10.1093/ons/opz294 31605114

[23] Wang Y Li P Zhang XJ Xu YY Wang W. Gamma knife surgery for cavernous sinus hemanginoma: a report of 32 cases. World Neurosurg. (2016) 94:18–25. 10.1016/j.wneu.2016.06.094 27373416

[24] Chibbaro S Cebula H Ganau M Gubian A Todeschi J Lhermitte B. Multidisciplinary management of an intra-sellar cavernous hemangioma: case report and review of the literature. J Clin Neurosci Off J Neurosurg Soc Aust. (2018) 52:135–8. 10.1016/j.jocn.2018.03.021 29622503

[25] Nakamura N Shin M Tago M Terahara A Kurita H Nakagawa K. Gamma knife radiosurgery for cavernous hemangiomas in the cavernous sinus. Report of three cases J Neurosurg. (2002) 97:477–80. 10.3171/jns.2002.97.supplement_5.0477 12507080

[26] Li ZH Wu Z Zhang JT Zhang LW. Surgical management and outcomes of cavernous sinus hemangiomas: a single-institution series of 47 patients. World Neurosurg. (2019) 122:e1181–e94. 10.1016/j.wneu.2018.11.015 30447442

[27] Chen CL Leu CH Jan YJ Shen CC. Intraventricular cavernous hemangioma at the foramen of Monro: case report and literature review. Clin Neurol Neurosurg. (2006) 108:604–9. 10.1016/j.clineuro.2005.04.004 15916846

[28] Yin YH Yu XG Xu BN Zhou DB Bu B Chen XL. Surgical management of large and giant cavernous sinus hemangiomas. J Clin Neurosci Off J Neurosurg Soc Aust. (2013) 20:128–33. 10.1016/j.jocn.2012.01.050 23164822

[29] Carrasco R Pedrosa M Pascual JM Navas M Liberal R Sola RG. Cavernous angiomas of the lateral ventricles. Acta Neurochir. (2009) 151:149–54. 10.1007/s00701-009-0186-8 19194650

[30] Andoh T Shinoda J Miwa Y Hirata T Sakai N Yamada H. Tumors at the trigone of the lateral ventricle: clinical analysis of eight cases. Neurol Med Chirurgica. (1990) 30:676–84. 10.2176/nmc.30.676 1708458

[31] Han MS Moon KS Lee KH Kim SK Jung S. Cavernous hemangioma of the third ventricle: a case report and review of the literature. World J Surg Oncol. (2014) 12:237. 10.1186/1477-7819-12-237 25069472 PMC4124769

[32] Sinson G Zager EL Grossman RI Gennarelli TA Flamm ES. Cavernous malformations of the third ventricle. Neurosurgery. (1995) 37:37–42. 10.1097/00006123-199507000-00005 8587688

[33] Katayama Y Tsubokawa T Maeda T Yamamoto T. Surgical management of cavernous malformations of the third ventricle. J Neurosurg. (1994) 80:64–72. 10.3171/jns.1994.80.1.0064 8271024

[34] Nannucci S Pescini F Poggesi A Ciolli L Patrosso MC Marocchi A. Familial cerebral cavernous malformation: report of a further Italian family. Neurol Sci. (2009) 30:143–7. 10.1007/s10072-009-0020-3 19184323

