# Supplementary material for: Surgical resection of intracranial cavernous hemangioma located at uncommon location: Clinical presentation and management
Source: Front Neurol. 2023 Feb 17;14:1105421. doi: 10.3389/fneur.2023.1105421 (PMC9981967; doi:10.3389/fneur.2023.1105421)
Supplement: Supplementary file 1 [file Table_1.docx]

Supplementary Table 1: Baseline characteristics of UCHs

| Patient | Age (years),  Sex | Duration  (months) | Symptom | Lesion location | Size  (mm) | Surgery,  EOR | Intraoperative bleeding  (ml) | GKRS | Outcome | KPS | Follow-Up  (months) |
| --- | --- | --- | --- | --- | --- | --- | --- | --- | --- | --- | --- |
| 1 | 33, F | 2 | Blurred vision, dizziness | Sellar and suprasellar region | 24 | Endo, PR | 800 | Yes | Alive | 70 | 103 |
| 2* | 14, F | 3 | Headache, dizziness | Parasellar region | 89 | Micro, STR | 3000 | Yes | Alive | 100 | 32 |
| 3 | 45, F | 8 days | Dizziness, headache | Parasellar and sellar region | 46 | Micro, STR | 1000 | No | Alive | 100 | 35 |
| 4 | 65, M | 1 | Dizziness, impaired vision | Sellar and parasellar region | 30 | Micro, PR | 800 | Yes | Alive | 80 | 120 |
| 5 | 46, M | 10 days | Headache | Sellar region | 22 | Endo, GTR | 100 | No | Alive | 90 | 139 |
| 6 | 63, F | 24 | Impaired vision | Suprasellar region | 46 | Endo, PR | 1200 | Yes | Alive | 60 | 127 |
| 7 | 34, M | 2 | Impaired vision | Suprasellar region | 20 | Micro, GTR | 150 | No | Alive | 70 | 64 |
| 8 | 43, F | 1 day | Headache, vomiting | Suprasellar region | 35 | Micro, GTR | 100 | No | Alive | 100 | 49 |
| 9 | 16, M | 2 | Headache, fever, vomiting | The septum pellucidum | 32 | Endo, GTR | 100 | No | Recurrence & de novo | 60 | 13 |
| 10 | 6, F | 2 | Weight gain | The third ventricle | 28 | Micro, GTR | 50 | No | Alive | 90 | 80 |
| 11† | 57, F | 3 | Headache, dizziness | Midbrain aqueduct | 25 | Endo, GTR | 20 | No | Alive | 100 | 12 |
| 12 | 45, M | 5 | Dizziness, headache, fever | Cerebral falx | 42 | Micro, GTR | 200 | No | Alive | 100 | 58 |
| 13 | 52, M | 2 weeks | Dizziness | Cerebral falx | 30 | Micro, GTR | 100 | No | Alive | 100 | 112 |
| 14* | 37, F | 1 | Sensory disturbance of right limbs | Parietal meninges | 50 | Micro, GTR | 150 | No | Alive | 100 | 79 |

*Endo* endoscopic surgical resection, *EOR* extent of resection, *F* female, *GKRS* gamma-knife radiosurgery, *GTR* gross total resection, *M* male, *Micro* microsurgical resection, *KPS* Karnofsky performance scale, *PR* partial resection, *STR* subtotal resection

*These two cases had been previously reported as single case report (6, 17).

†This case had been previously reported in Neurosurgical Focus Video (18).
