# Supplementary material for: Surgical resection of intracranial cavernous hemangioma located at uncommon location: Clinical presentation and management
Source: Front Neurol. 2023 Feb 17;14:1105421. doi: 10.3389/fneur.2023.1105421 (PMC9981967; doi:10.3389/fneur.2023.1105421)
Supplement: Supplementary file 2 [file Table_2.DOCX]

Supplementary Table 2: Radiological features of UCHs

| Patient | T1-weighted image | T2-weighted image | Gd-enhanced MRI | Initial diagnosis |
| --- | --- | --- | --- | --- |
| 1 | Hypo | Hyper | Homo | Meningioma |
| 2* | Hypo | Hyper | Hetero | Meningioma |
| 3 | Hypo | Hyper | Hetero | Meningioma |
| 4 | Hypo | Hyper | Hetero | Angioma |
| 5 | Hypo-hyper | Hypo-hyper | Hetero | Pituitary adenoma |
| 6 | Hypo | Hyper | Hetero | Craniopharyngioma |
| 7 | Hyper | Hypo | Hetero | Craniopharyngioma |
| 8 | Hypo-iso | Iso-hyper | Hetero | Cavernous hemangioma |
| 9 | Hypo-hyper | Hypo-hyper | Hetero | Cavernous hemangioma |
| 10 | Hypo-hyper | Hypo-hyper | Hetero | Cavernous hemangioma |
| 11† | Hypo-iso | Hypo-iso | Hetero | Cavernous hemangioma |
| 12 | Hypo | Hyper | Homo | Meningioma |
| 13 | Hypo | Hyper | Homo | Meningioma |
| 14* | Hypo | Hyper | Hetero | Meningioma |

hetero heterogeneously enhanced, homo homogeneously enhanced, hyper hyperintensity, Hypo hypointensity, iso isointensity

*These two cases had been previously reported as single case report (6, 17).

†This case had been previously reported as Video article in Neurosurgical Focus (18).
